# Anti-N-Methyl-D-Aspartate Receptor Encephalitis, an Underappreciated Disease in the Emergency Department

**DOI:** 10.5811/westjem.2016.2.29554

**Published:** 2016-05-02

**Authors:** Daniel R. Lasoff, Jimmy Corbett-Detig, Rebecca Sell, Matthew Nolan, Gabriel Wardi

**Affiliations:** * VA Medical Center, San Diego, California; † UCSD Medical Center, Department of Emergency Medicine, Division of Medical Toxicology, San Diego, California; ‡ UCSD Medical Center, Department of Emergency Medicine, San Diego, California; § UCSD Medical Center, Department of Internal Medicine, Division of Pulmonary, Critical Care, and Sleep Medicine, San Diego, California

## Abstract

Anti-N-Methyl-D-Aspartate Receptor (NMDAR) Encephalitis is a novel disease discovered within the past 10 years. Antibodies directed at the NMDAR cause the patient to develop a characteristic syndrome of neuropsychiatric symptoms. Patients go on to develop autonomic dysregulation and often have prolonged hospitalizations and intensive care unit stays. There is little literature in the emergency medicine community regarding this disease process, so we report on a case we encountered in our emergency department to help raise awareness of this disease process.

## CASE REPORT

A 23-year-old man presented to the emergency department (ED) after a witnessed tonic-clonic seizure. He was previously healthy with no prior seizures and had no recent trauma, fevers, vomiting, or history of substance abuse except for marijuana. His family reported over the preceding weeks he had been trying to lose weight and recently had started using three weight-loss supplements: Erratic, Thermovex, and Prozein. A review of these supplements revealed they were a mixture of various amino acids, proteins, vitamins, and caffeine. He had reportedly been agitated and increasingly manic over the previous few days, and co-workers stated that he had seemed confused at work earlier in the day. A review of systems was otherwise negative. On physical examination, the patient was a young, athletic male who was somnolent but arousable. His temperature was 37.1°C, blood pressure 132/71, heart rate 62bpm, and respiratory rate 16 breaths per minute. Pupils were 4mm, equal, round, and reactive. His face was symmetric and tongue was midline on protrusion. He had 5/5 strength in both upper and lower extremities, and sensation was intact throughout to fine touch. Patellar and ankle reflexes were 2+, symmetric and without clonus. Laboratory analysis was remarkable for a glucose level of 232mg/dL, a urine drug screen that was positive for THC and benzodiazepines (the latter of which had been given by the field medics and the ED for seizures). Following an unremarkable computed tomography (CT) of his brain, he had a lumbar puncture, which showed 370 white blood cells/mm^3^, 300 red blood cells/mm^3^, and a protein level of 147mg/dL. The gram stain of his cerebrospinal fluid (CSF) was negative. Empiric ceftriaxone, vancomycin, and acyclovir were started for presumed infectious meningitis. While in the ED, the patient had an additional tonic-clonic seizure and was intubated for airway protection. The patient was then admitted to the intensive care unit (ICU) for further care. During his course in the ICU he failed to improve, remained intermittently agitated and was unable to be extubated. An electroencephalogram (EEG) shortly after admission revealed ongoing epileptiform activity, and he received aggressive anti-seizure therapy. Consultations from infectious disease and rheumatology services were unable to provide a diagnosis. Autoimmune panels and several viral, fungal, and bacterial assays were all negative. On hospital day 15, the patient’s CSF was sent for an anti-N-Methyl-D-Aspartate receptor (NMDAR) antibody assay, and the test returned two days later with a titer of 1:5120 (normal <1:10) consistent with anti-NMDAR encephalitis. The patient was started on intravenous immunoglobulin (IVIG) for treatment, followed by plasmapheresis, cyclophosphamide, and eventually rituximab. His course has been complicated by episodes of autonomic instability, delirium, and hospital-associated infections. He remains in the ICU six months after admission for management of severe autonomic instability and remains dependent upon a tracheostomy and gastrostomy tube.

## DISCUSSION

Anti-NMDAR encephalitis is an autoimmune encephalitis syndrome that is underappreciated and frequently missed in the ED due to lack of awareness. It was initially described in 2007 by Dalmau et al., and to our knowledge, it has not received any attention in the US emergency medicine literature to date. It is one of a growing family of neuronal surface antibody syndromes (NSAS) with auto-antibodies directed against the NR1 subunit of the NMDA-receptor.^1^,^2^ As awareness grows, it seems that anti-NMDAR encephalitis is likely to be four times more common than HSV encephalitis.^3^ Our goal is to increase awareness of anti-NMDAR encephalitis as many patients initially present to the ED with classic histories for this condition, but the diagnosis is not considered until much later in the clinical course.

The diagnosis is often difficult to make due to the nonspecific nature of symptoms.^4^ Anti-NMDAR encephalitis classically presents with a prodromal syndrome of malaise, headache, and fever followed by psychiatric symptoms such as irritability, agitation, hallucinations, memory loss, mania, or frank psychosis.^5^–^8^ Neurological symptoms such as aphasia, seizures, dyskinesias, catatonia, or coma distinguish this syndrome from a pure psychiatric illness.^2^,^5^–^7^ Patients frequently develop autonomic dysregulation as well, which can manifest as tachycardia, hyperthermia, hypothermia, blood pressure abnormalities, or hypoventilation which frequently necessitates mechanical ventilation.^2^,^5^–^7^

Due to the nonspecific presenting symptoms, patients will often undergo lengthy workups, repeated imaging and blood work, and several consultations from specialists, without a clear diagnosis. Symptoms are frequently attributed incorrectly to a toxicological or psychiatric cause.^5^,^9^ In Dalmau et al.’s 2008 case series of 100 patients, 77 were initially seen by a psychiatrist.^5^ Due to the often-varied presenting symptoms, diagnoses on average were delayed 21 days in children and 28 days in adults from the time of symptom onset.^10^ This is quite concerning as patient outcome seems to worsen when treatment is delayed.^10^

Anti-NMDAR encephalitis is more prevalent in women.^3^,^11^ Patients tend to be young with ages ranging from 2–40.^3^,^4^ The autoantibodies can be associated with paraneoplastic syndromes in 20–59% of cases, most commonly ovarian teratomas.^2^,^5^–^7^,^10^ Males and young children are less likely to have an underlying tumor that is responsible for their encephalitis.^5^,^11^ In cases associated with a tumor, patients may improve with removal of the tumor.^13^

Patients with undifferentiated encephalitis typically undergo evaluation with neuroimaging, lumbar puncture, and EEG. Patients with anti-NMDAR encephalitis may show nonspecific abnormalities on MRI, although the majority are normal.^5^,^7^ Almost all patients will have nonspecific EEG changes such as delta waves, theta waves, or slowing, and about half of patients may show epileptiform activity.^5^ CSF analysis is also non-specific, but common findings include lymphocytic pleocytosis, increased protein, and increased opening pressures; however, a significant number of patients present without any of these findings.^7^,^14^ The hallmark of the disease is the presence of anti-NMDAR antibodies that can be found in the both the serum and the CSF. The CSF appears to be more sensitive, as one study showed all 43 patients were positive for antibodies in the CSF, but only 27 patients tested positive for antibodies in their serum.^14^

First-line therapy for anti-NMDAR encephalitis includes steroids, IVIG, and plasma exchange.^10^,^11^ Patients who do not respond to first-line therapy have been treated with immunomodulators such as cyclophosphamide and rituximab.^10^,^15^ If there is evidence of ovarian teratomas, operative removal may be beneficial, and complete cessation of symptoms has been reported.^10^,^16^ While the therapy for anti-NMDAR encephalitis will almost certainly not be started in the ED, the consideration of and appropriate diagnostic testing for this condition will greatly aid these patients.

Unfortunately, many patients with anti-NMDAR encephalitis tend to undergo prolonged hospitalizations and require lengthy ICU stays. The mortality seems to be lower than initially thought and is estimated at approximately 10%; the majority of patients make a meaningful neurologic recovery. 1,10 It also appears that early treatment results in a better neurologic outcome for patients.^18^

## CONCLUSION

Anti-NMDAR encephalitis carries a significant morbidity and mortality that is worsened by delays in diagnosis; it is underappreciated and unrecognized in the ED This case illustrates some common features of anti-NMDAR encephalitis: a patient with recent psychiatric symptoms who presents with a neurological complaint, a delay in diagnosis and a lengthy course of treatment. Although our patient fits the usual age range and had common presenting symptoms of behavioral changes and seizures, anti-NMDAR encephalitis is more common in women. Emergency physicians who encounter patients with new onset neurologic complaints preceded by psychiatric symptoms should consider anti-NMDAR encephalitis in the differential diagnosis as it may promote earlier treatment and improve outcomes.

## References

[1] Zuliani L, Graus F, Giometto B (2012). Central nervous system neuronal surface antibody associated syndromes: review and guidlines for recognition. J Neurol Neurosurg Psychiatry.

[2] Dalmau J, Gleichman AJ, Hughes EG (2008). Anti-NMDA-receptor encephalitis: case series and analysis of the effects of antibodies. Lancet Neurol.

[3] Gable MS, Sheriff H, Dalmau J (2012). The frequency of autoimmune N-Methyl-D-Aspartate receptor encephalitis surpasses that of individual viral etiologies in young individuals enrolled in the California Encephalitis Project. Clin Infect Dis.

[4] Barry H, Byrne S, Barrett E (2015). Anti-N-methyl-D-aspartate receptor encephalitis: review of clinical presentation, diagnosis and treatment. BJPsych Bull.

[5] Irani SR, Bera K, Waters P (2010). N-methyl-D-aspartate antibody encephalitis: temporal progression of clinical and paraclinical observations in a predominantly non-paraneoplastic disorder of both sexes. Brain.

[6] Dalmau J, Tuzun E, Wu HY (2007). Paraneoplastic anti-N-methyl-D-aspartate receptor encephalitis associated with ovarian teratoma. Ann Neurol.

[7] Florance NR, Davis BA, Dalmau J (2009). Anti-N-Methyl-D-Asparate receptor (NMDAR) encephalitis in children and adolescents. Ann Neurol.

[8] Hughes EG, Peng X, Gleichman AJ (2010). Cellular and synaptic mechanisms of anti-NMDA receptor encephalitis. J Neurosci.

[9] Punja M, Pomerleau AC, Devlin JJ (2013). Anti-N-methyl-D-aspartate receptor (anti-NMDAR) encephalitis: an etiology worth considering in the differential diagnosis of delirium. Clin Toxicol (Phila).

[10] Titulaer MJ, McCracken L, Gabilondo I (2013). Treatment and prognostic factors for long-term outcome in patients with anti-NMDA receptor encephalitis: an observational cohort study. Lancet Neurol.

[11] Ramanathan S, Mohammad SS, Brilot F (2014). Autoimmune encephalitis: Recent updates and emerging challenges. J Clin Neurosci.

[12] Dalmau J, Lancaster E, Martinez-Hernandez E (2011). Clinical experience and laboratory investigations in patients with anti-NMDAR encephalitis. Lancet Neurol.

[13] Seki M, Suzuki S, Iizuka T (2008). Neurological response to early removal of ovarian teratoma in anti-NMDAR encephalitis. J Neurol Neurosurg Psychiatry.

[14] Wang R, Guan HZ, Ren HT (2015). CSF findings in patients with anti-N-methyl-D-aspartate receptor-encephalitis. Seizure.

[15] Liba Z, Sebronova V, Komarek V (2013). Prevalence and treatment of anti-NMDA receptor encephalitis. Lancet Neurol.

[16] Iizuka T, Sakai F, Ide T (2008). Anti-NMDA receptor encephalitis in Japan Long-term outcome wtihout tumor removal. Neurology.

[17] Peery HE, Day GS, Dunn S (2012). Anti-NMDA receptor encephalitis. The disorder, the diagnosis, and the immunobiology. Autoimmun Rev.

[18] Finke C, Kopp UA, Pruss H (2012). Cognitive Deficits following anti-NMDA receptor Encephalitis. J Neurol Neurosurg Psychiatry.

